# Global assessment of the fate of nitrogen deposition in forest ecosystems: Insights from ^15^N tracer studies

**DOI:** 10.1515/biol-2025-1171

**Published:** 2025-09-18

**Authors:** Xinlu Bai, Yaping Li, Jinhu Zhi

**Affiliations:** Agricultural College of Tarim University, Alar, 843300, China; Biogas Institute of Ministry of Agriculture and Rural Affairs, Chengdu, 610041, China; Horticulture and Forestry College of Tarim University, Alar, 843300, China

**Keywords:** nitrogen deposition, nitrogen uptake, nitrogen retention, nitrogen losses, controlling factor

## Abstract

Atmospheric nitrogen (N) deposition is recognized as a pivotal nutrient input in forest ecosystems. However, significant gaps persist in our comprehension of the global-scale fate of N in forest ecosystems. In a pioneering effort, this study analyzed the fluxes and determinants of deposited N by 234 observations from 52 published articles. Our findings indicated that plant uptake, soil retention, and N losses, respectively, accounted for 27.4, 57.9, and 14.6% of the total deposited N. The fate of deposited N was significantly influenced by a suite of factors, including forest type, climatic parameters such as mean annual temperature (MAT) and precipitation (MAP), edaphic characteristics such as soil pH and the carbon to nitrogen ratio (C/N), and experimental factors like nitrogen addition rate (NR), nitrogen forms (NF), plot size (PS) for ^15^N studies, and the duration of study. For the uptake of deposited N, MAP emerged as the predominant positive factors, whereas NR was the dominant negative factors; for deposited N soil retention, NR was the key positive factors, while MAT was the key negative factors; for N losses, MAP was the predominant positive factors, with the C/N ratio serving as a significant negative factor. Thus, for a given forest ecosystem with relatively stable climate and soil conditions, NR, NF, and the soil C/N were the main controlling factors regulating the fate of deposited N. These insights significantly advance our grasp of the N cycle in forest ecosystems. Consecutive monitoring of the impact of deposited N on soil N transformations and carbon sequestration is needed in future studies.

## 1 Introduction

Human activities, such as fossil fuel consumption, animal husbandry expansion, and agricultural intensification, have strongly accelerated both reactive nitrogen (N) emissions and their deposition in the terrestrial environment globally [1,2]. By the end of the twentieth century, the rate of nitrogen deposition had at least doubled [3], with a substantial amount destined for terrestrial ecosystems. Increased N deposition can alter N cycles, affect ecosystem functions [4,5], and increase N losses via leaching and denitrification, especially in forest ecosystems, because of its limited N availability [6,7,8]. Critically, the effects of N deposition on forest ecosystems depends on the fate of deposited N, including uptake by plants [9,10], retention in the soil [11], and losses [12]. Distinguishing the fate of the deposited N is crucial to understand the effects of N deposition on forest ecosystems better. Many studies have been conducted to assess the deposited N flux by ^15^N addition experiments at the field scale [13,14]. Xie et al. [15] and Leeson et al. [16] found that N deposition significantly increased ammonia (NH_3_) volatilization, nitrous oxide (N_2_O) emissions, and nitrate leaching in forest ecosystems. The accumulation of N in the soil and plant bodies increases with the N deposition [17,18,19]. In addition, determining the fate of deposited N is also essential for an in-depth study of carbon sequestration in forest systems, as the relationship between carbon and nitrogen is very close [20,21,22]. Therefore, it is essential to determine the fate of deposited N in forest systems on a global scale. However, identifying the distribution of deposited N on a global scale remains challenging, due to the challenges of complexity, laboriousness, and the expensive cost of the ^15^N addition experiments method [23,24].

The fate of deposited N can be influenced by various factors, including forest type (FT), climate (e.g., mean annual air temperature and precipitation), forms of N input (e.g., \[{\text{NH}}_{4}^{+}]\] vs \[{\text{NO}}_{3}^{-}]\] ), and soil properties [25]. Wang et al. [26] revealed that tropical forests accumulate more ^15^N in mineral soil and plants than temperate forests because of their greater biomass and faster N transfer from organic soil to mineral soil. Veerman et al. [12] reported that soil carbon stock, organic layer thickness, and mean annual temperature (MAT) are the controlling factors for ^15^N retention in the organic soil layer. In contrast, soil carbon stock, pH, moisture content, and soil bulk density are the controlling factors for ^15^N retention in the mineral soil layer. Liu et al. [27] and Wang et al. [26] identified the different fates of deposited \[{\text{NH}}_{4}^{+}]\] and \[{\text{NO}}_{3}^{-}]\] in temperate and Asian tropical primary forests, respectively. Previous studies have evaluated the controlling factors for deposited N at specific experimental sites. However, these sites are often constrained by factors such as geographical location, climate conditions, and soil types, which significantly limit their representativeness. Therefore, it is difficult to comprehensively understand the fates of deposited N in forest ecosystems, without clarifying the relative roles of influencing factors on a global scale. Meta-analysis quantitatively integrates data from numerous independent studies, synthesizing dispersed findings into robust conclusions with enhanced statistical power and generalizability. This approach provides reliable, efficient evidence for large-scale scientific decisions, enabling a global assessment of deposited N fate and examination of interactions among FTs, climate, soil properties, and experimental conditions [28].

In an effort to bridge the existing gaps in knowledge, this study is designed to quantify the dynamics and fate of N deposits in forest ecosystems. The objectives of the present study are (1) to determine the fate of deposited N in forest ecosystems on a global scale by meta-analysis based on ^15^N tracer studies and (2) to determine the controlling factors influencing deposited N fate in forest ecosystems.

## 2 Materials and methods

### 2.1 Data collection and dataset overview

**Figure 1:**
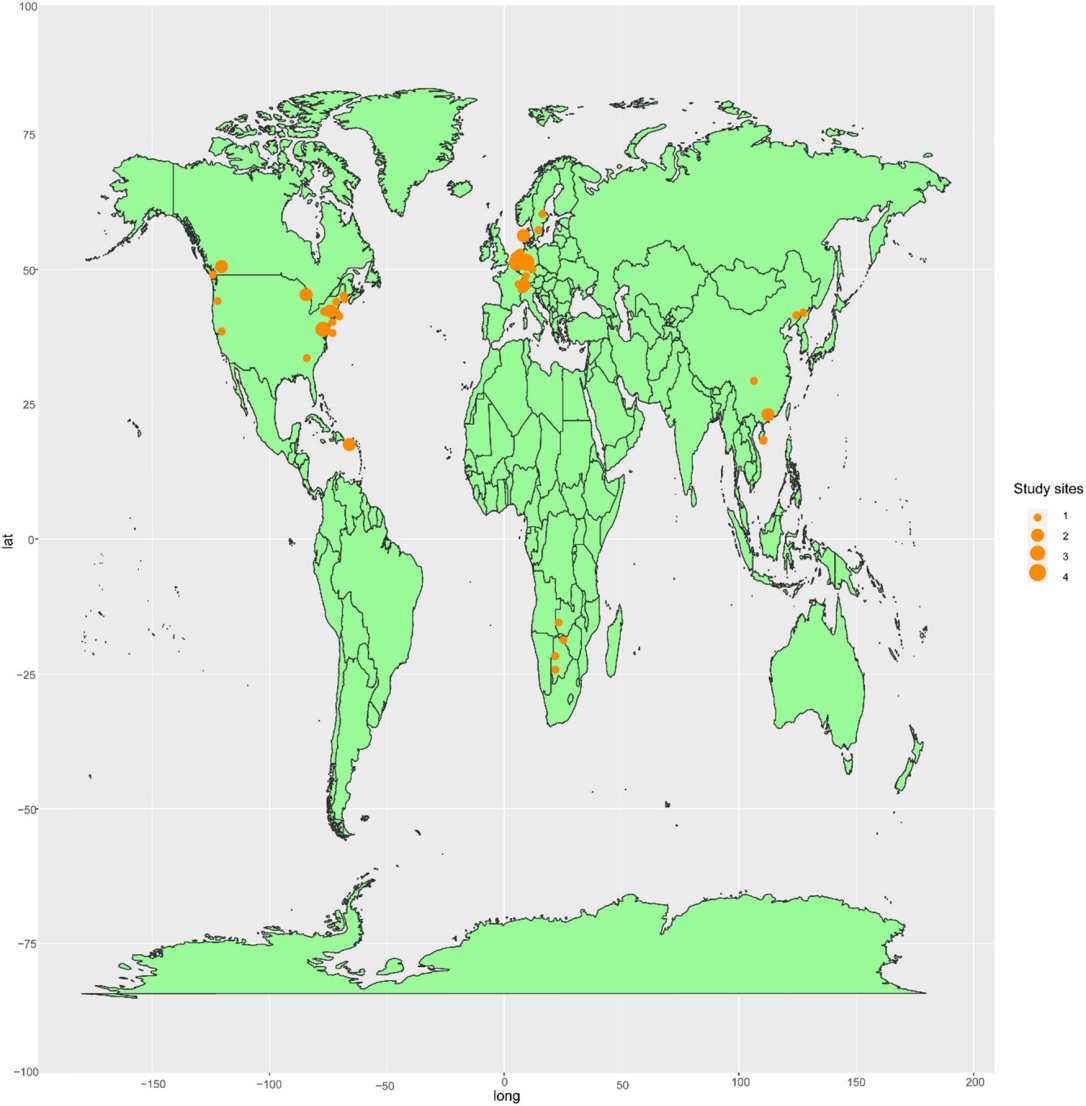
Global distribution of study sites included in this work.

The dataset was generated by collecting 234 observations from 52 papers containing data from ^15^N tracer studies across various forest ecosystems. We used the following terms: “the fate of deposited N”; “fates of atmospheric deposited N”; “different fates of deposited N”; “retention of deposited N”; “pathways and dynamics of deposited N” and “recovery of deposited N” to search for publications in Google Scholar and the Web of Science Database up to 31 March, 2024. We used the following criteria for collecting data: (1) conducted the study via field experiments rather than soil column and pot experiments and (2) measured N fluxes using the ^15^N tracer method. Data were obtained from tables and figures. If the data were presented only in figures, they were digitized using Get-Data v.2.22 (http://gatdata-graph-digitizer.com). The site-specific details were assembled to analyze the patterns and controlling factors of the fate of the deposited N. The experimental location, FT, MAT, mean annual precipitation (MAP), N addition rate (NR), N forms (NF), soil pH, soil carbon to nitrogen ratio (C/N), ^15^N plot size (PS), and study duration time (SDT) were assembled. We matched these environmental factors with ^15^N uptake, ^15^N retention, and ^15^N losses (Table 1). The experimental locations are shown in Figure 1.

**Table 1:** Characteristics of the climatic variables (MAT, MAP), soil properties (pH, C/N), and study variables (PS, SDT, and NR) from the peer-reviewed articles

Terms	MAT (°C)	MAP (mm)	pH	C/N	PS (m^2^)	SDT (year)	NR (kg N ha^−1^)
Range	−5.4 to 25.6	365–5,500	3.0–8.0	11.6–64.0	0.03–3,000	0.04–23	0.03–640
Mean value	8.4	1,405	4.6	24.3	136	2.8	49.1
SD	5.1	1,003	1.3	10.6	125	3.2	32.9
*n*	87	92	73	67	121	102	202

MAT, mean annual temperature; MAP, mean annual precipitation; C/N, the ratio of soil carbon to nitrogen; PS, 15N plot size; SDT, study duration time; NR, nitrogen rate; NF, nitrogen forms. “SD” and “*n*” represent the standard deviation and number of all observations.

### 2.2 Data analyses

Prior to analysis, all data were checked for normality using the Shapiro–Wilk test. The 95% confidence intervals (CIs) were generated using bootstrapping (4,999 iterations). We calculated the relative importance of the explanatory variables, defined as the magnitude of the increase in the mean squared errors of the multiple linear regression models when one of the selected explanatory variables was randomly permuted, to determine the controlling factors influencing the deposited N fates [29]. Multiple linear regression models were also used [30]: *Y* = *β*_0_ + *β*_1_ × *X*_1_ + *β*_2_ × *X*_2_ + *β*_3_ × *X*_3_ + *β*_4_ × *X*_4_ + *β*_5_ × *X*_5_ + *β*_6_ × *X*_6_ + *β*_7_ × *X*_7_ + *β*_8_ × *X*_8_ + *ε*, where *β*_0_, *β*, and *ε* are the intercept, slope value, and sampling error, respectively; and *X*_1_, *X*_2_, *X*_3_, *X*_4_, *X*_5_, *X*_6_, *X*_7_, and *X*_8_ refer to MAT, MAP, NR, NF, soil pH, soil carbon to nitrogen ratio, ^15^N PS, and duration time of the study, respectively. The relative importance of the explanatory variables was obtained using relative weights via the “Car” and “Mass” packages R software [31]. Specifically, this method quantifies the independent contribution of each explanatory variable to the response variable by decomposing the model’s coefficient of determination (*R* ^2^). This approach circumvents the bias induced by multicollinearity among predictor variables, which is a common issue in traditional regression analysis. Relative weight analysis provides a more precise reflection of each variable’s importance ranking within the overall model, particularly in scenarios involving highly correlated predictors. The multiple linear regression models and associated predictor variables are presented in Table 2.

**Table 2:** Models of multiple linear regression on the fate of deposited nitrogen and associated predictor variables

Fate	Predictor variables	Estimate	Standard error	*p* value	Significance level	*R* ^2^	Adjusted *R* ^2^
Uptake	MAT	−0.57	0.27	*p* < 0.01	**	0.603	0.581
	MAP	0.01	0.01	0.03	*		
	PS	0.01	0.01	0.13			
	SDT	1.01	0.31	0.21			
	pH	−0.24	0.04	0.16			
	C/N	0.15	0.05	0.04	*		
	NR	−0.31	0.11	0.02	*		
	NF	0.11	0.03	0.12			
	Intercept	15.74	7.84	0.04	*		
Retention	MAT	−0.97	0.33	*p* < 0.01	**	0.565	0.463
	MAP	0.01	0.01	0.14			
	PS	0.01	0.01	0.15			
	SDT	−0.16	0.06	0.23			
	pH	2.39	0.84	0.03	*		
	C/N	0.05	0.01	0.03	*		
	NR	0.03	0.01	0.02	*		
	NF	−0.09	0.02	*p* < 0.01	**		
	Intercept	66.45	8.78	*p* < 0.01	**		
Losses	MAT	2.01	0.61	*p* < 0.01	**	0.732	0.654
	MAP	0.03	0.01	0.12			
	PS	0.03	0.01	0.11			
	SDT	0.28	0.23	0.12			
	pH	0.63	0.47	0.82			
	C/N	0.30	0.12	0.02	*		
	NR	0.03	0.01	0.02	*		
	NF	0.30	0.21	0.55			
	Intercept	−7.51	2.32	0.02	*		

MAT, mean annual temperature; MAP, mean annual precipitation; C/N, the ratio of soil carbon to nitrogen; PS, ^15^N plot size; SDT, study duration time; NR, nitrogen rate; NF, nitrogen forms.

## 3 Results

### 3.1 Nitrogen uptake

**Figure 2:**
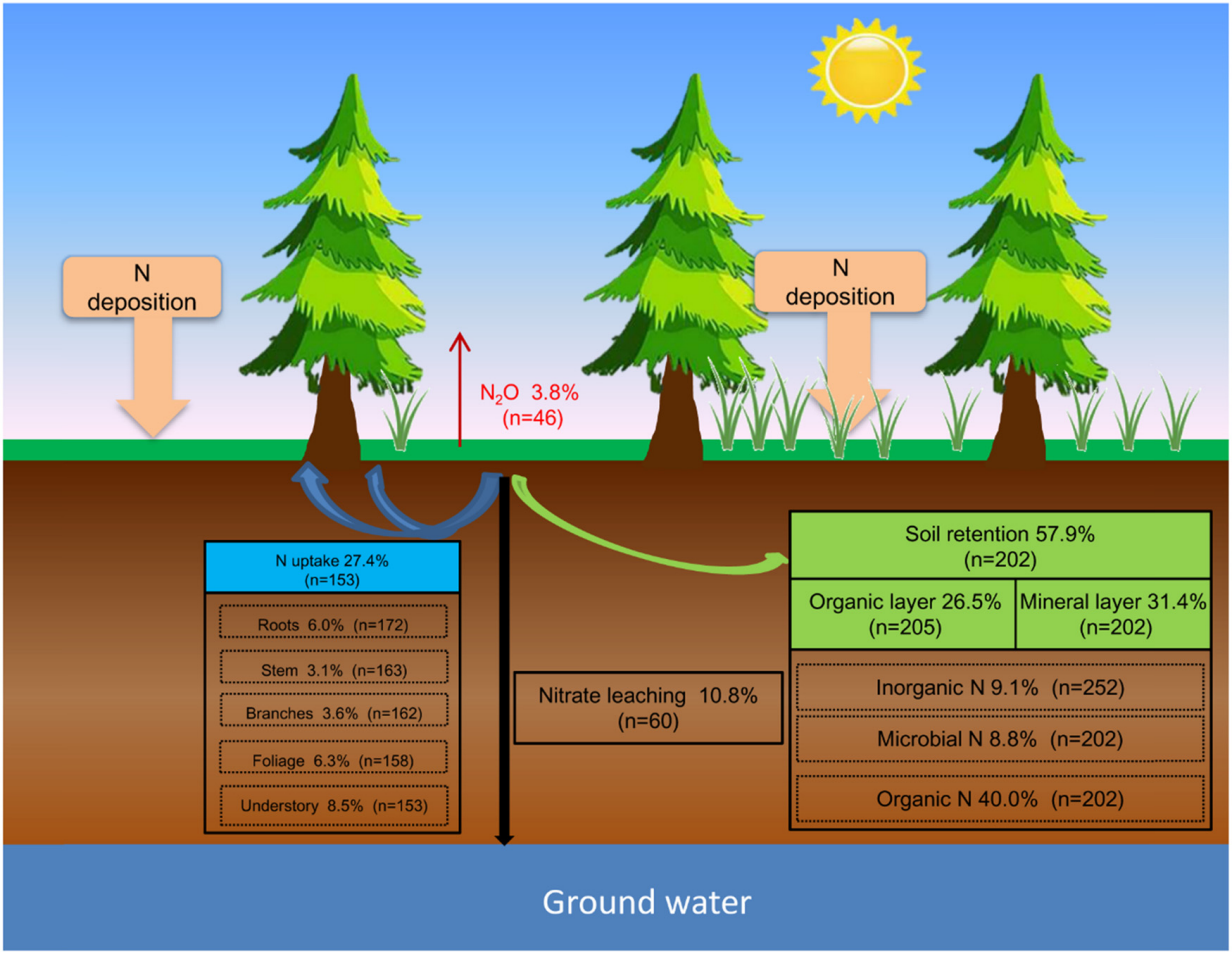
Fate of the deposited N in forest ecosystems. The numbers in the parentheses are the numbers of observations.

Total N uptake accounted for 27.4% of the deposited N, and the roots, stems, branches, foliage, and understory accounted for 6.0, 3.1, 3.6, 6.3, and 8.5% of the total N uptake, respectively (Figure 2). FT strongly influenced N assimilation, with tropical/subtropical evergreen forests (TSFs) exhibiting the highest uptake capacity, followed by temperate broad-leaved (TBF) and coniferous forests (TCF). Moreover, it had higher N uptake with warm temperatures, low precipitation, and low soil pH and C/N. Warmer temperatures and intermediate-to-high precipitation enhanced N uptake. The amounts of N uptake decreased as follows: 31.6% (MAT > 10°C), 26.3% (6°C ≤ MAT ≤ 10°C), 24.0% (MAT < 6°C); 23.9% (MAP ≤ 1,000 mm), 29.7% (1,000 mm < MAP < 1,500 mm), 32.4% (MAP ≥ 1,500 mm); 34.3% (pH ≤ 4), 28.7% (4 < pH ≤ 6), 24.9% (pH > 6); and 34.2% (C/N ≤ 20), 26.7% (20 < C/N ≤ 30), and 22.1% (C/N > 30).

**Figure 3:**
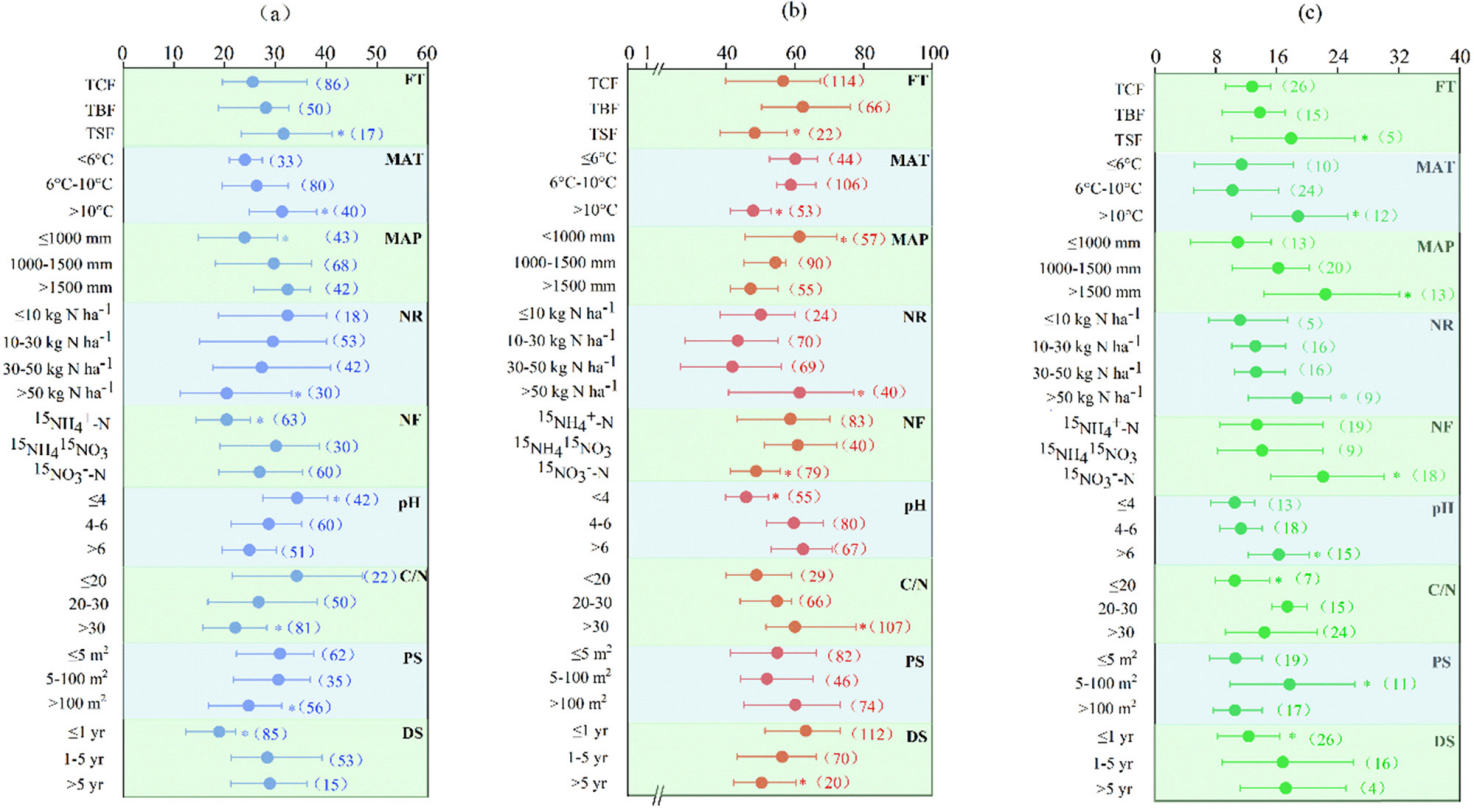
Effect of FT, MAT, MAP, NR, NF, soil pH, soil carbon to nitrogen ratio, ^15^N PS, and SDT on nitrogen uptake (a), nitrogen retention (b), and nitrogen losses (c), respectively. FT, forest type; TCF, temperate coniferous forest; TBF, temperate broad-leaved forest; TSF, tropic and subtropical evergreen forest; MAT, mean annual temperature; MAP, mean annual precipitation; NR, deposited nitrogen rate; NF, deposited nitrogen forms; C/N, the ratio of soil carbon to nitrogen; PS, 15N plot size; and SDT, study duration time. The numbers in the parentheses are the number of observations. The values are mean values with 95% CIs. *Correlation is significant at *p* < 0.05.

In addition to FT, climate, and soil properties, the characteristics of deposited N and experimental duration also contributed to the differences in N uptake. Compared with the low NR (NR ≤ 50 kg N ha^−1^), the high NR (NR > 50 kg N ha^−1^) significantly decreased the total N uptakes by 26.4–38.0%. The total N uptake was 20.4, 30.1, and 26.9% when applied as ^15^ \[{\text{NH}}_{4}^{+}]\] −N, ^15^NH_4_^15^NO_3_, and ^15^ \[{\text{NO}}_{3}^{-}]\] -N, respectively. In addition, the total N uptake was 30.9, 30.6, and 24.7% when the PS was <5 m^2^, 5–100 m^2^, and > 100 m^2^, respectively. The total N uptake was 18.9, 28.4, and 28.9% when the experiment was continued for <1, 1–5, and >5 years, respectively (Figure 3a).

### 3.2 Nitrogen retention

N retention in the soil was dominated and accounted for 57.9% of the deposited N, including 26.5% in the organic layer and 31.4% in the mineral layer. Microbial N, inorganic N, and organic N accounted for 8.8, 9.1, and 40.0% of residual N, respectively (Figure 2). The N retention in TCPs and TBFs was 56.6 and 62.3%, respectively, which was significantly higher than that in TSFs (48.3%). The climate also contributed to differences in N retention. The N retention was 58.8–60.1% with MAT ≤ 10°C and 61.3% with MAP ≤ 1,000 mm, which were significantly higher than those of 47.9% MAT > 10°C and 47.1–54.3% with MAP > 1,000 mm, respectively (Figure 3b).

Large amounts of deposited N led to more residual N in the soil. The N retention was 61.4% with NR > 50 kg N ha^−1^, which was significantly higher than that of 41.8–50.1% with NR < 50 kg N ha^−1^. For deposited N forms, \[{\text{NO}}_{3}^{-}]\] -N deposition introduced the lowest N retention of 48.7%, relative to 58.7% for ^15^ \[{\text{NH}}_{4}^{+}]\] -N deposition and 60.8% for ^15^NH_4_^15^NO_3_ deposition, respectively. In addition, significantly higher N accumulation was observed in soils with a pH > 4 and C/N > 30. The N retention was 63.2 and 56.3% when the experiment continued for <1 and 1–5 years, respectively, which was significantly higher than 50.3% when the experiment continued for >5 years (Figure 3b).

### 3.3 Nitrogen losses

The N loss accounted for 14.6% of the deposited N, including 10.8% for nitrate leaching and 3.8% for nitrous oxide emission, respectively (Figure 2). The total N losses were 12.8 and 13.8% in TCPs and TBFs, respectively, and significantly increased by 29.7–39.8% in TSFs, respectively. Total N losses were also dependent on climate and soil conditions. The highest N losses occurred with MAT > 10°C and MAP > 1,500 mm. Moreover, our meta-analysis showed that soil pH and C/N ratio positively affected total N loss. The total N losses were significantly higher with soil pH > 6 (16.3%), and with soil C/N ≥ 20 (17.4 and 14.4%), respectively.

In addition, the characteristics of deposited N also contributed to the differences in N losses. Total N losses were 9.3, 13.2, 12.3, and 15.7% with deposited N < 10 kg N ha^−1^, 10–30 kg N ha^−1^, 30–50 kg N ha^−1^, and > 50 kg N ha^−1^, respectively. Total N losses were much more pronounced for ^15^ \[{\text{NO}}_{3}^{-}]\] -N deposition (22.1%), followed by ^15^NH_4_^15^NO_3_ deposition (14.1%) and ^15^ \[{\text{NH}}_{4}^{+}]\] -N deposition (13.4%). Furthermore, the ^15^N PS and study duration significantly affected N loss. The total N losses were significantly higher with ^15^N PS of 5–100 m^2^ (17.7%), and with a study duration of more than 1 year (16.8–17.2%) (Figure 3c).

### 3.4 Explanatory factors for the fate of deposited N

**Figure 4:**
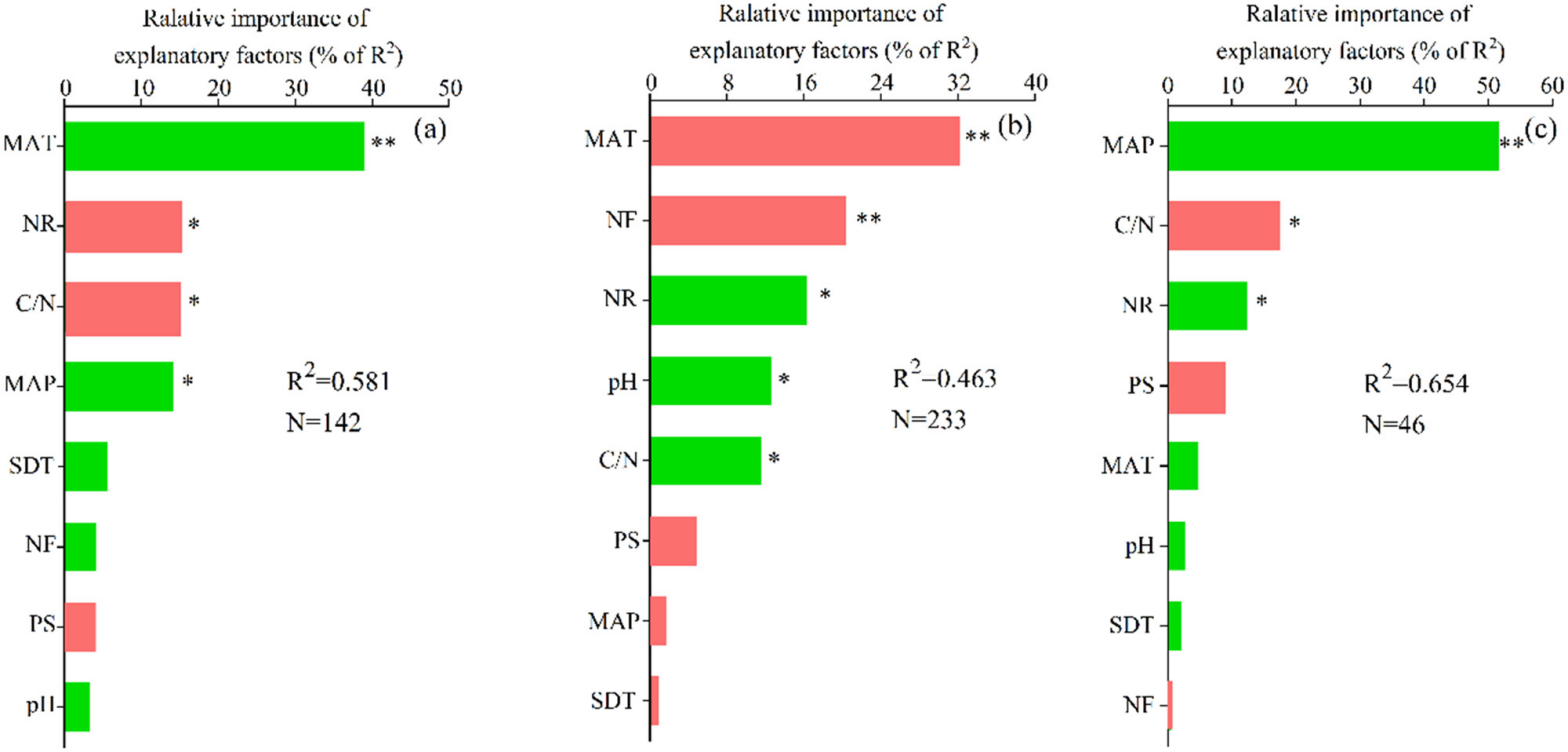
Relative importance of explanatory factors for nitrogen uptake (a), nitrogen retention (b), and nitrogen losses (c) in forest ecosystems. Green columns indicate a positive effect, and red columns indicate a negative effect. ** and *** represent significance at *p* < 0.05 and *p* < 0.01, respectively. The predictor variables of multiple linear models are described in Table 1. MAT, mean annual temperature; MAP, mean annual precipitation; NR, nitrogen rate; NF, nitrogen form; C/N, the ratio of soil carbon to nitrogen; PS, ^15^N plot size; SDT, study duration time.

Deposited N uptake significantly increased with MAT and MAP, whereas significantly decreased with NR and soil C/N (Figure S1). MAT (38.9% of *R* ^2^) and MAP (14.1% of *R* ^2^) were the dominant positive factors and the deposited N rate (NR, 15.1% of *R* ^2^) and C/N (14.9% of *R* ^2^) were the dominant negative factors for deposited N uptake in forest ecosystems. Deposited N retention significantly increased with NR, soil pH, and C/N, whereas significantly decreased with MAT (Figure S2). NR (16.2% of *R* ^2^), pH (12.6% of *R* ^2^), and C/N (11.6% of *R* ^2^) were the dominant positive factors, whereas MAT (32.1% of *R* ^2^) and the deposited N form (NF, 20.3% of *R* ^2^) were the dominant negative factors for deposited N retention in forest ecosystems. In addition, deposited N losses significantly increased with MAP and NR, whereas significantly decreased with C/N (Figure S3). MAP (51.6% of *R* ^2^) and NR (12.3% of *R* ^2^) were the dominant positive factors, and C/N (17.4% of *R* ^2^) was the dominant negative factor for deposited N losses in forest ecosystems (Figure 4).

## 4 Discussion

### 4.1 Differential fates of deposited N in forest ecosystems

The total deposited N recoveries for plant and soil pools were 80–88% in forest ecosystems (Figure 3), which is consistent with previous studies. Gurmesa et al. [32] reported that 66–68% of the deposited N was retained in plant and soil pools. Templer et al. [33] reported that more than 70% of the deposited N was retained in plant and soil pools. N retention in the soil dominated and shared 60–69% of the total N recovery. Similarly, a new study by Sun et al. [34] showed that 84% of deposited N was retained in the heavily polluted forests of the Beijing-Tianjin-Hebei region. Considering that forest soil has a high C/N ratio and large amounts of easily available carbon [35], it is a strong sink for N mobilization. In addition, the results revealed that a greater recovery of deposited N was observed in TBF and TCP than in TSF because of higher N retention in soil. These results, indicate that lower N losses were responsible for greater N retention in the soil of TBF and TCP.

Nitrate leaching shared 70–81%, and dominated N losses in forest ecosystems (Figure 3). Similarly, Fang et al. [36] and Liu et al. [37] reported similar nitrate leaching rates in forest ecosystems. Nitrate leaching is also significantly influenced by precipitation [38,39]. Therefore, significantly higher nitrate leaching and total N loss occurred in TSFs. In addition, losses via N_2_O emissions accounted for only a small proportion of the total deposited N, which is consistent with previous studies. Eickenscheidt et al. [40] reported N_2_O emission of 0.3–2.6 kg N ha^−1^ year^−1^ in beech and spruce forests. In addition, ammonia volatilization and denitrification losses represent major mechanisms for nitrogen loss from deposited N. However, current research on ammonia volatilization and denitrification losses associated with deposited N remains relatively scarce, with incomplete data and unclear mechanisms, making it challenging to effectively incorporate these processes into the analysis. Therefore, we suggest that subsequent research should focus on the dynamic mechanisms, influencing factors, and quantitative analysis of ammonia volatilization and denitrification processes in deposited N. This will enhance our understanding of deposited N loss pathways and provide a scientific basis for relevant environmental management and emission reduction strategies.

### 4.2 Controls on fates of deposited N in forest ecosystems

The results of our analyses demonstrated that various factors influence the fate of deposited N in forest ecosystems. MAT and MAP were the dominant positive factors (53.1% of *R* ^2^) for deposited N uptake. MAT and MAP significantly affect forest plant growth, and suitable temperatures and precipitation increase forest plant biomass and, therefore, deposited N uptake. The results revealed that NR and C/N were the dominant negative factors (30.0% of *R* ^2^) for deposited N uptake. Because forest ecosystems are commonly considered N-deficient, more deposited N could be expected to be absorbed with increased deposited N [22,40]. However, lower rates of deposited N were absorbed with a high deposited N load, which was supported by our results showing that deposited N uptake rates significantly decreased with deposited N > 50 kg N ha^−1^ (Figure 3). Similarly, Templer et al. [33] reported that N uptake rates decrease with deposited N, with an apparent threshold rate of 46 kg N ha^−1^ year^−1^. When the deposited N rate in forest ecosystems exceeds 50 kg N ha⁻¹ year⁻¹ (which is far beyond the global critical load threshold of 5–25 kg N ha⁻¹ year⁻¹), the N retention capacity collapses [41]. The core mechanism underlying this phenomenon lies in the imbalance of multi-level biogeochemical processes. The deposited N is predominantly in the form of \[{\text{NH}}_{4}^{+}]\] . High concentrations of \[{\text{NH}}_{4}^{+}]\] / \[{\text{NO}}_{3}^{-}]\] exert feedback inhibition on key enzymes involved in N assimilation, leading to the accumulation of free amino acids. Meanwhile, excessive deposited N input induces nutrient deficiencies (e.g., deficiencies in phosphorus and magnesium when the N:P ratio exceeds 20) and inhibits mycorrhizal symbiosis, thereby weakening the root absorption capacity [42]. Deposited N input reduces the C:N ratio of litter (to <20:1), accelerating the depletion of organic carbon, triggering carbon limitation, and reducing N fixation efficiency. High \[{\text{NH}}_{4}^{+}]\] concentrations promote nitrification, increasing \[{\text{NO}}_{3}^{-}]\] production and leaching losses. Along with \[{\text{NO}}_{3}^{-}]\] leaching, base cations are also lost in large quantities. This process exacerbates soil acidification and aluminum toxicity, disrupting the nitrogen cycle [43]. In addition, C/N is an important indicator of soil C and N availability, and soils with high C/N ratios are likely to contribute to relatively high C availability for microbes, which could promote microbial N immobilization and reduce N uptake by forest plants [44]. NR, pH, and C/N ratio were the dominant positive factors (40.4% of *R* ^2^) for deposited N retention. Deposited N provides a substantial substrate for N accumulation in the soil; therefore, N retention significantly increases with deposited N rate. Soil pH is another primary determinant of deposited N retention. The richness and diversity of microorganism are significantly higher in alkaline and neutral soils than in acidic soils [45,46,47]. Thus, increasing the soil pH increased microbial activity and N immobilization, significantly. As mentioned above, a high soil C/N ratio is favorable for deposited N immobilization and accumulation in the soil. MAT and NF were the dominant negative factors (52.4% of *R* ^2^) for deposited N retention. Precipitation is a key factor affecting the solute transport process of N. Large amounts of precipitation are responsible for more N leaching and runoff losses, while less N remains residual in the soil [48,49]. Moreover, soil aeration conditions depend on precipitation, and increasing precipitation leads to increased N_2_O emissions and reduced N retention in the soil [50,51]. The recovery of N in the soil was negatively correlated with N electron valence, suggesting that deposited ammonium is retained in the soil more proportionally than nitrate. The greater recovery of ammonium in the soil can be attributed to the preferential assimilation and retention of ammonium by microbes and abiotic immobilization, such as soil adsorption [52,53]. \[{\text{NH}}_{4}^{+}]\] directly enters the plant cytoplasm via ammonium transporters on the plasma membrane through passive diffusion or proton gradient-driven symport. This process does not rely on ATP hydrolysis for energy. During microbial immobilization, \[{\text{NH}}_{4}^{+}]\] is directly incorporated into the glutamine synthetase-glutamate synthase cycle, demonstrating significantly higher metabolic efficiency in carbon skeleton utilization compared to the dissimilatory nitrate reduction to ammonium pathway. Consequently, ammonium nitrogen exhibits higher assimilation priority in both plant and microbial systems. Additionally, the acidic soil environments predominant in forest ecosystems effectively suppress ammonia volatilization losses of ammonium nitrogen, further enhancing its ecological retention efficiency. MAP and NR were the dominant positive factors (63.9% of *R* ^2^) for deposited N loss. In general, deposited N provides a substantial substrate for N loss; therefore, N losses significantly increase with deposited N. Similarly, previous studies have highlighted the exponential relationship between N inputs and nitrate leaching, nitrous oxide, nitric oxide, and ammonia volatilization [54]. In addition, the increased N losses with MAP could be attributed to suitable conditions for N losses under heavy precipitation. The C/N ratio was the dominant negative factor (17.4% of *R* ^2^) for deposited N loss. Specifically, a sufficient carbon supply activates microbial synthesis of N containing enzymes, enabling the rapid conversion of inorganic N into microbial biomass N and stable organic N polymers [55]. Microbes secrete extracellular enzymes (e.g., chitinases and proteases) in the rhizosphere, which accelerate the mineralization of organic nitrogen. However, the microbial community promptly re-assimilates the mineralized nitrogen products through a process termed the “microbial loop.” This leads to a sharp decline in inorganic nitrogen concentrations in the rhizosphere, with reductions reaching up to 50–80% [56].

### 4.3 Implications and suggestions

The intergovernmental panel on climate change concluded that climate change was no longer a subject of debate. Climate change manifests itself in many ways, including increased temperatures, changes in precipitation, and precipitation trends. The results of our analyses demonstrated that climate change could significantly affect the fate of deposited N in forest ecosystems. The positive correlation between MAT and deposited N uptake (Figure 4a) was consistent with the result of Templer et al. [33] and Elrys et al. [57]. The pattern of greater deposited N uptake with higher MAT suggested that higher temperatures may have led to higher rates of N uptake by plants [58]. However, greater deposited N uptake could not offset the possible increases in the rates of soil N-cycling processes, such as mineralization and nitrification, and losses via gas emissions or leaching [10]. Therefore, the total ecosystem recovery (N uptake and N retention in the soil) significantly decreased (Figure 3). These results agreed with the watershed mass balances, showing that N exports increase with increasing temperatures [59,60].

Precipitation also controls the fate of deposited N in forest ecosystems. The N retention in the soil was negatively correlated with MAP, whereas the N loss was positively correlated with MAP (Figure 3). These results suggested that N export increased with increasing precipitation. N export from soils may increase soil CO_2_ efflux into the atmosphere, thereby affecting the global carbon cycle and contributing to global warming [61,62]. Therefore, consecutive monitoring of soil N retention, carbon sequestration, and CO_2_ emissions is required for future studies.

Global N deposition is likely to increase in the future, increasing N loss (Figure 4). Recently, Tang et al. [63] revealed that increased N deposition stimulated tropical montane forest soil N_2_ and N_2_O emissions. Moreover, the increased N deposition causes soil acidification [64], thereby influencing the soil N cycling processes.

The form of deposited N has changed, particularly in China, where the growth rate of deposited nitrate was the highest [65]. Although the total ecosystem N retention was similar for ammonium and nitrate, plants took up more nitrate, and the soil retained more ammonium (Figure 3). This suggested that increased deposited Nitrate could lead to N export from soils, and thereby influencing soil N and C cycles. Furthermore, nitrate is vulnerable to leaching losses, and nitrate leaching deteriorates as more nitrogen is deposited in the form of nitrate. Therefore, the consecutive monitoring of nitrate leaching is required in future studies to better understand the relationships between deposited N and water pollution.

## 5 Conclusion

Our study provides a comprehensive evaluation of deposited N in forest ecosystems. Our findings revealed that plant uptake, soil retention, and N loss were 27.4, 57.9, and 14.6%, respectively. Deposited N uptake was positively affected by MAT and MAP, but negatively affected by the deposited N rate and C/N. Soil retained deposited N was positively affected by deposited N rate, soil pH, and C/N, but negatively affected by MAT and deposited N form. Deposited N losses were positively affected by MAP and deposited N rate, but negatively affected by C/N. We also found that changes in climate and deposited N could significantly influence soil N export and thereby, soil carbon sequestration. Thus, consecutive monitoring of soil N retention and carbon sequestration dynamics is critically needed, not merely to document biogeochemical fluxes, but to quantify the trade-offs between nitrogen export and ecosystem services.

## Supplementary Material

Supplementary Figure
