# Supplementary material for: Global assessment of the fate of nitrogen deposition in forest ecosystems: Insights from 15N tracer studies
Source: Open Life Sci. 2025 Sep 18;20(1):20251171. doi: 10.1515/biol-2025-1171 (PMC12451427; doi:10.1515/biol-2025-1171)
Supplement: Supplementary Figure [file biol-2025-1171-sm.pdf]

Supplementary material

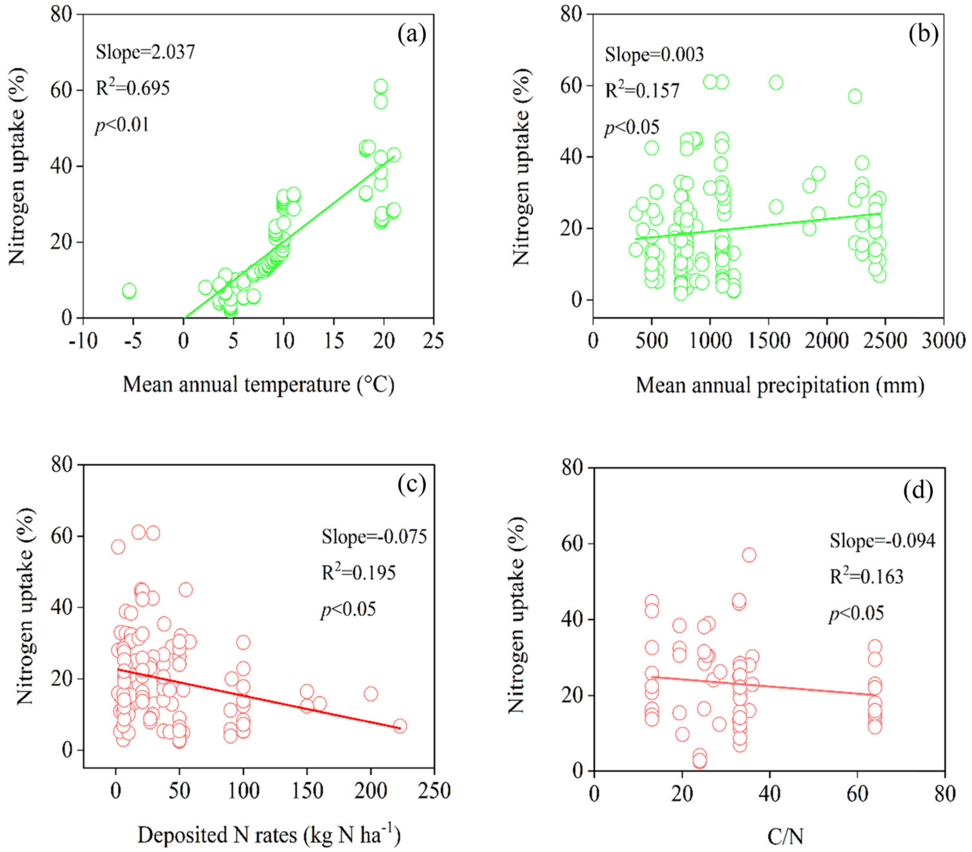

Figure S1: The relationships between mean annual temperature and nitrogen uptake (a), mean annual precipitation and nitrogen uptake (b), deposited nitrogen rates and nitrogen uptake (c), and C/N and nitrogen uptake (d), respectively.

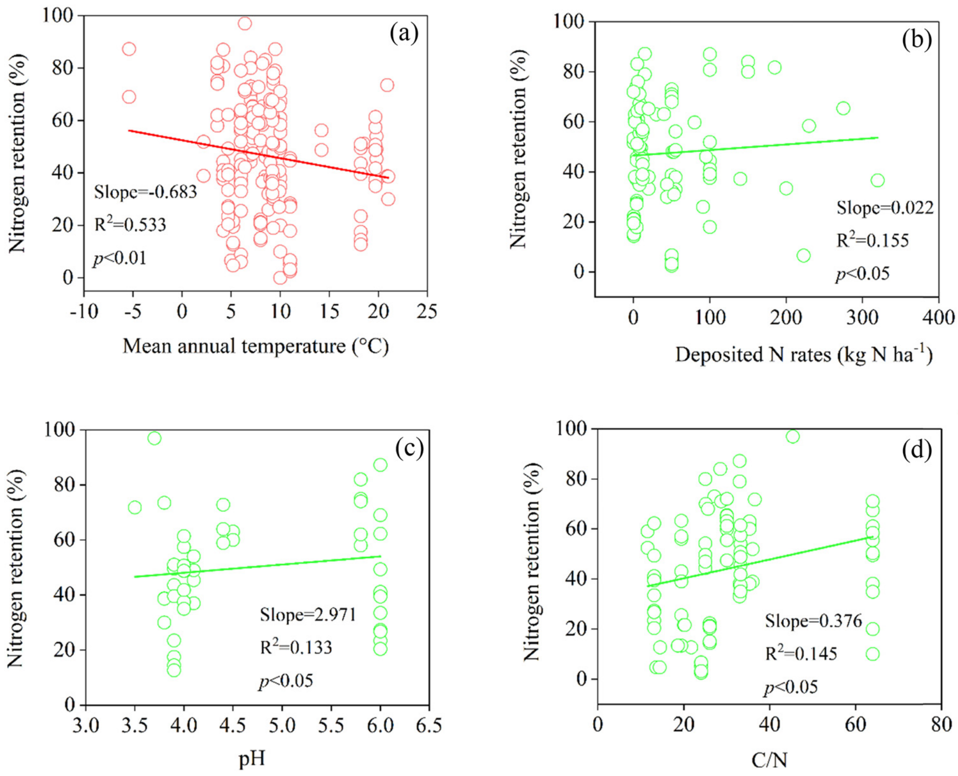

Figure S2: The relationships between mean annual temperature and nitrogen retention (a), deposited nitrogen rates and nitrogen retention (b), pH and nitrogen retention (c), and C/N and nitrogen retention (d), respectively.

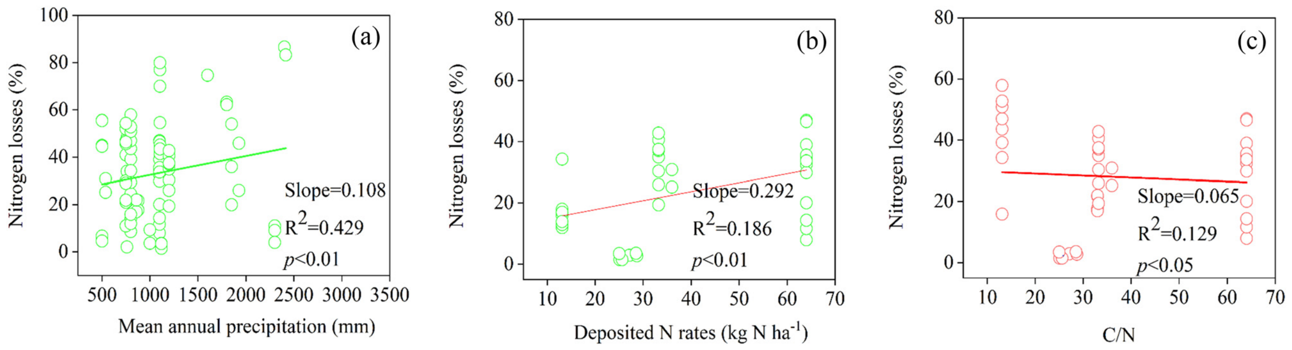

Figure S3: The relationships between mean annual precipitation and nitrogen losses (a), deposited nitrogen rates and nitrogen losses (b), C/N and nitrogen losses (c), respectively.
